# The integrated effect of salinity, organic amendments, phosphorus fertilizers, and deficit irrigation on soil properties, phosphorus fractionation and wheat productivity

**DOI:** 10.1038/s41598-020-59650-8

**Published:** 2020-02-17

**Authors:** Zheli Ding, Ahmed M. S. Kheir, Marwa G. M. Ali, Osama A. M. Ali, Aly I. N. Abdelaal, Xin’e Lin, Zhaoxi Zhou, Bizun Wang, Beibei Liu, Zhenli He

**Affiliations:** 1 Haikou Experimental Station, Chinese Academy of Tropical Agricultural Sciences (CATAS), Haikou, China; 2 Soils, Water and Environment Research Institute, Agricultural Research Center, Giza, Egypt; 3 Crop Science Department, Faculty of Agriculture, Menoufia University, Shebin El-Kom, Egypt; 4 Institute of Environmental and Plant Protection/Environmental Impact Assessment and Risk Analysis Center, Chinese Academy of Tropical Agricultural Sciences, Haikou, Hainan China; 5 University of Florida, Institute of Food and Agricultural Sciences, Indian River Research and Education Center, Fort Pierce, FL 34945 USA

**Keywords:** Plant sciences, Environmental sciences, Solid Earth sciences

## Abstract

Soil degradation due to global warming, water scarcity and diminishing natural resources negatively impacts food security. Soil fertility deterioration, particularly phosphorus (P) deficiency, remains a challenge in the arid and semi-arid regions. In this study, field experiments were conducted in different geographical locations to investigate the effects of organic amendments coupled with P fertilization and irrigation on soil physical-chemical properties, and the growth, yield and quality of wheat. Application of P fertilizers combined with organic amendments mitigated soil salinity, increased organic matter content, available water, hydraulic conductivity and available macronutrients, but decreased soil bulk density. Application of organic amendments slightly increased total Cd, Ni and Pb in soil, but Cd and Ni concentration was below allowable limits whilst Pb reached a hazardous level. Soil P fractions were significantly increased with the combined application of mineral P and organic amendments irrespective of salinity and irrigation. Crop growth yield and quality of wheat improved significantly in response to the integrated application of mineral P and organic amendments. In conclusion, the combination of mineral P sources with organic amendments could be successfully used as a cost-effective management practice to enhance soil fertility and crop production in the arid and semi-arid regions stressed with water scarcity and natural resource constraints.

## Introduction

Saline soils are an important natural resource but the area of degraded saline soils worldwide has rapidly increased due to climate change and limited rainfall, which poses a great challenge to global food security^1,2^. This problem may be solved through a targeted remediation program of such soils. Deficit irrigation (DI) is also projected to increase soil salinity and sodicity, particularly in the arid and semi-arid climatic regions^3^, requiring proper management strategies to alleviate soil degradation. Contamination of soils with heavy metals has become a global concern, due to potential hazardous impacts of these elements on soil quality, crop yield and quality^4^, and food safety and human health^5^. Application of organic amendments was reported to remediate saline soils, alleviate salinity and sodicity stress on crops^6^, and reduce toxicity of heavy metals^4^. Some organic amendments contain heavy metals, and their application benefits require further studies^7^. Organic amendments could improve soil properties by accelerating leaching of sodium and other salts and reducing exchangeable sodium percentage (ESP)^8^. Moreover, organic amendments enhance soil biological and enzyme activities and increase organism abundance, thus improving soil fertility and crop production^9^. However, more research is needed to explore effect of organic amendments combined with irrigation regimes on saline and alkaline soils. Phosphorus (P) is a major essential element for plant growth and production^10^. Due to rapid population expansion worldwide, the projected amount of P fertilizers will be doubled to enable sustainable food production^2^. Arid soils contain much less available P, as compared to humid regions, due to low total P and high fixation of P in soils^11^. In the arid region low water inputs limit salt leaching, resulting in accumulation of calcium (Ca) minerals^12^. Most of water soluble P applied in fertilizers to soils is rapidly converted to other forms with less availability^13^. Chemical fractionation of soil P could provide further information about P mobility and availability in soils^14^. However, there is no further investigation for determining the effect of P fertilizers on soil P fractionation at a field scale, particularly in the arid and semi-arid ecosystems. Given this situation, there is an urgent need for more efficient use of P, while reducing the environmental impacts of mineral fertilizers^15^. This could be achieved through the integration between P mineral fertilizers and organic amendments, which requires more attention. Various types of organic amendments have been used to remediate saline soils, some of them untreated and inexpensive such as poultry manure^16^, crop straw and factory residues^17^, farmyard manure (FYM)^18^, and sewage sludge (SS)^8^. The SS is reported as a valuable fertilizer for wheat, increasing soil organic matter content^7^, which requires further evaluation in field. Furthermore, integration between soil amendments and irrigation in saline soils is not well studied. Unequivocally, phosphate fertilizers could improve crop production^19^ and soil microbial activity^15^, but limited studies focused on saline alkaline soils. Exploring the integrated effects of phosphate fertilizers, deficit irrigation and organic amendments on saline soil quality and crop production is imperative for managing soils in the arid and semiarid regions. The main objectives of this study were: (I) to explore the combined effect of phosphate fertilizers (superphosphate and ammonium phosphate), organic amendments (farmyard manure and sewage sludge) and irrigation regime (full and deficit) on soil properties, P fractionation, and heavy metal accumulation (Cd, Ni and Pb), (II) to explore the relationship between soil salinity, organic matter, available P, applied irrigation water and soil P fractionation, and (III) to investigate the effect of different treatments on wheat productivity and quality traits on three typical soil types (non-saline, saline and highly saline).

## Results

### Soil chemical and physical properties at three different locations

**Figure 1:**
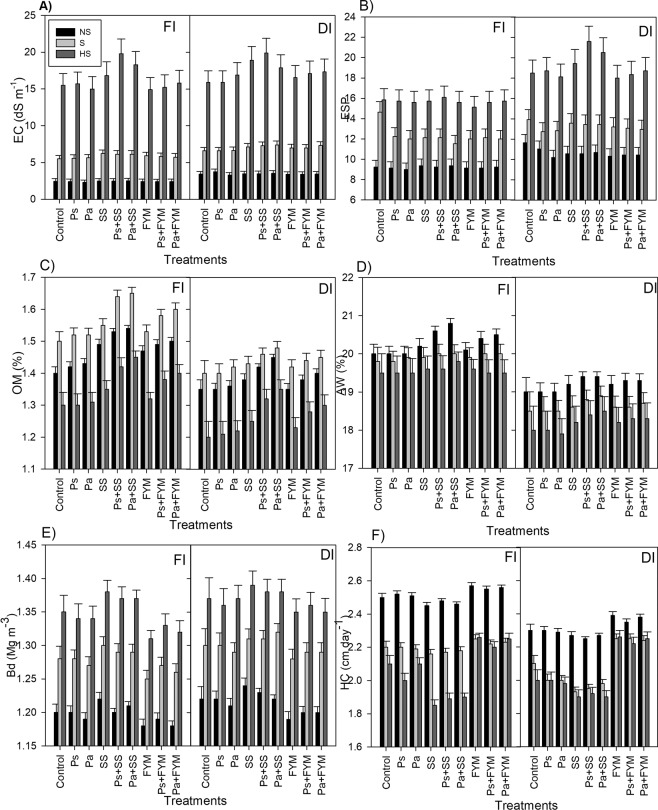
Soil salinity (**A**), sodicity (**B**), organic matter (**C**), available water (**D**), bulk density (**E**) and hydraulic conductivity (**F**) as affected by irrigation regime full (FI), deficit irrigation (DI), phosphorus fertilizers and their mixtures with organic amendments. Phosphorus fertilizers included single superphosphate (Ps) and diammonium phosphate (Pa), organic amendments included farmyard manure (FYM) and sewage sludge (SS). All treatments were conducted in three soils with different salinity levels (i.e. non-saline soil, N-S; saline soils, S and highly saline soils, HS). Bars represent average values after two successive growing seasons, error bars represent standard deviation (n = 3).

Soil in three locations differed in salinity content, which in turn, caused variations in other physical, chemical and nutritional properties such as pH, ESP, OM, bulk density (Bd), HC and available macronutrients (Supplementary Table S1 and Fig. 1). Application of P fertilizers and organic amendments influenced these properties. Soil pH effect was slightly but significantly decreased by application of SS and FYM in all soils compared to P fertilizers and control treatments (Supplementary Table S1). The lowest values of pH were recorded when SS added followed by FYM treatment. There were no significant differences between P fertilizers and control on soil pH. SS alone or combined with P fertilizers led to the highest values of soil salinity (Fig. 1A). This effect was more profound in highly saline soil than saline or non-saline soils. P fertilization slightly increased EC, more with superphosphate (Ps) than ammonium phosphate (Pa), whereas application of FYM alone resulted in the lowest values of soil salinity, but a slight increase in EC occurred if combined with P fertilizers (Fig. 1A). As for irrigation regime, DI increased soil salinity by 13%, as compared to FI (Fig. 1A). Application of FYM and P fertilizers as well as FI slightly decreased soil ESP, but SS and DI treatments increased these values (Fig. 1B). Soil organic matter content (OM) increased significantly in response to FYM and SS application and slightly increased with P fertilizer treatments (Fig. 1C). SS and Pa combination was superior to FYM and Ps for improving soil organic matter. Deficit irrigation and soil salinity significantly reduced soil OM under all the treatments, whilst a significant increase occurred with FI treatment (Fig. 1C). Soil available water (AW) as a function of water holding capacity in response to soil properties was more affected by organic amendment than P fertilizers (Fig. 1D). The higher values of AW were observed when SS or FYM was added. There was no significant effect of P fertilizers on soil AW for all the studied locations. Increasing soil salinity decreased AW regardless of treatments (Fig. 1D). Consequently, soil physical (Bd and HC) and chemical properties (Fig. 1A–C) were significantly changed under the treatments of irrigation, P fertilization or organic amendment (Fig. 1E,F). SS alone or combined with P fertilizers (SS + Ps, and SS + Pa) significantly increased soil Bd (Fig. 1E), but decreased soil HC (Fig. 1F). Treatments with FYM, FYM + Ps, or FYM + Pa showed an opposite trend, decreasing soil Bd and increasing soil HC. Application of Ps or Pa alone had no significant effects on soil Bd and HC. Soil Bd values increased with increasing soil salinity, with the highest value occurring in the highly saline soil, followed by saline and non-saline soils (Fig. 1E). The lowest value of HC was recorded in the highly saline soil while the highest value occurred in the non-saline soil (Fig. 1F). Deficit irrigation slightly increased soil Bd but decreased HC, and vice versa with FI treatment.

### Soil macronutrients and heavy metal content at three different locations

**Figure 2:**
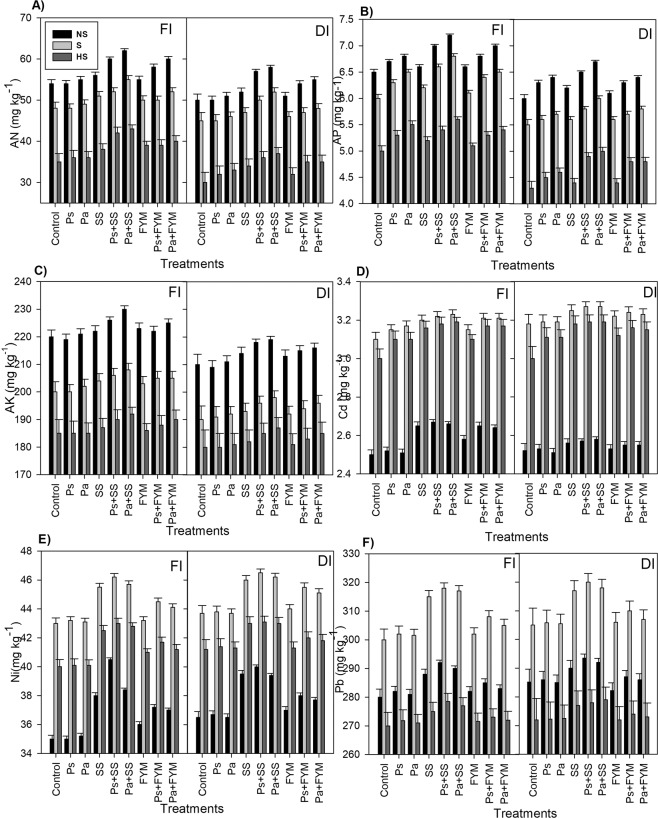
Soil available N (**A**), P (**B**) and K (**C**), as well as total cadmium (**D**), Nickel (**E**) and lead **(F**) as affected by irrigation regime full (FI), deficit irrigation (DI), phosphorus fertilizers and their mixtures with organic amendments. Phosphorus fertilizers included single superphosphate (Ps) and diammonium phosphate (Pa), organic amendments included farmyard manure (FYM) and sewage sludge (SS). All the treatments were conducted in three soils with different salinity levels (i.e. non-saline soil, N-S; saline soils, S, and highly saline soils, HS, down). Bars represent average values after two successive growing seasons, error bars represent standard deviation (n = 3).

Soil available macro-nutrients (N, P and K) significantly increased due to organic amendments and P fertilization (Fig. 2A–C). The increase varied among the treatments and decreased in the order of Pa + SS > Ps + SS > Pa + FYM > Ps + FYM > SS > FYM > Pa > Ps, indicating that SS and Pa treatment is most effective in improving soil macronutrients. Furthermore, these nutrients were higher under FI than DI treatment, irrespective of soils and amendments. Increasing soil salinity decreased the availability of soil macronutrients, leading to the lowest values of N, P and K in the highly saline soils (Fig. 2A–C). Application of organic amendment (SS or FYM) alone, or combined with P fertilizers (Ps + SS, Pa + SS, Ps + FYM and Pa + FYM) increased soil contamination by Cd, Ni, or Pb (Fig. 2D–F). DI treatment resulted in higher contents of toxic metals (Cd, Ni and Pb) than FI treatment regardless of soil type and amendments. Interestingly, higher contents of Cd and Ni were observed in saline soils followed by highly saline soil and non-saline soils, respectively (Fig. 2D,E). Meanwhile, the lowest value of Pb metal was recorded in the highly saline soil (Fig. 2F).

### Soil P fractionation

**Figure 3:**
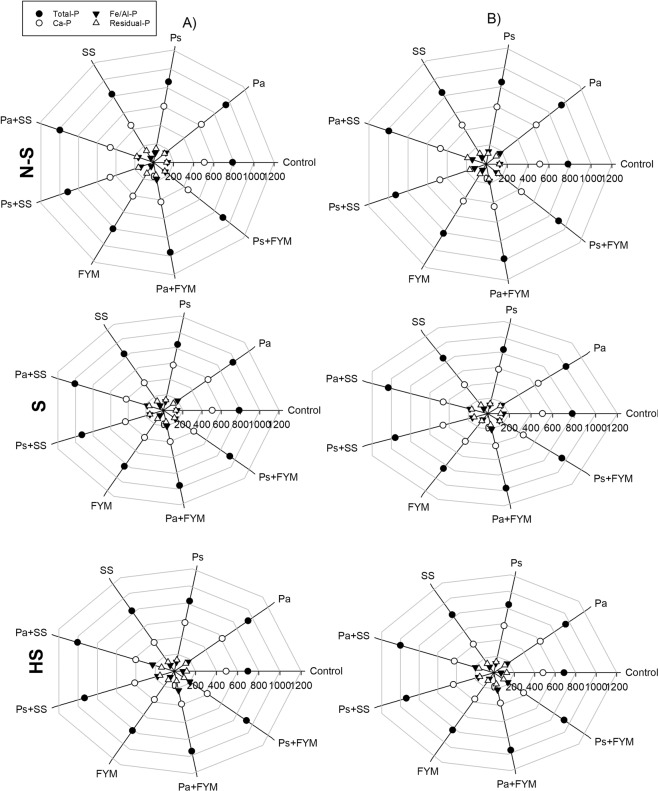
Radar plots of soil P fractions as affected by P fertilizers, super phosphate (Ps), diammonium phosphate (Pa), organic amendments (sewage sludge, SS and farmyard manure, FYM) as well as combination of P fertilizers with organic amendments under different irrigation regimes (full, A and deficit, B) in three soils with different salinity levels (non-saline, N-S; saline, S and highly saline, HS).

Changes in soil P fractions in response to different amendments, soil salinity and irrigation regime are shown in Fig. 3. Total P contents increased due to P fertilization and organic amendment for all the soils. The highest values were obtained when soil was treated with organic amendments combined with P fertilizers (Ps + SS, Pa + SS, Ps + FYM or Pa + FYM). Application of organic amendments alone resulted in lower values of total P, as compared with treatments with mineral P fertilizers (Fig. 3). Soil salinity slightly decreased total P fractions under all the treatments, leading to the lowest values of total P in the highly saline soil. The effect of irrigation regime on soil total P was non-significant, despite a slight decrease of total P in DI treatment compared with FI (Fig. 3). Calcium bound P (Ca-P) was the most abundant fraction (Fig. 3). In contrast to total P (Fig. 3) and available P (Fig. 2B), Ca-P decreased after application of organic amendments (Fig. 3). The largest decrease in this fraction occurred in the treatment of SS or FYM. Treatments with P mineral fertilizers (Ps or Pa) increased Ca-P fraction. Salinity and irrigation had minimal effect on this fraction, despite a slight decrease in case of DI and higher salinity. Compared to Ca-P fraction, Fe/Al -P fraction increased with application of organic amendment or mineral P fertilizers (Fig. 3). This fraction decreased with increasing soil salinity and with decreasing irrigation. As considered the most stable and recalcitrant fraction, residual-P was the second most abundant fraction. The highest values of residual-P were recorded when soil was treated with Ps + SS or Pa + SS. Furthermore, application of mineral P fertilizers alone or combined with FYM increased residual-P fraction in all the soils. Salinity and deficit irrigation slightly decreased this fraction (Fig. 3).

### Wheat growth, yield and quality subjected to soil salinity, irrigation regime, P fertilizers and organic amendments

Wheat phenology (anthesis and maturity dates) and plant height parameters were significantly promoted in response to application of amendments in all the studied soils (Table 1). Deficit irrigation treatment significantly reduced anthesis date, maturity date and plant height by 2.7, 4.5 and 10.7%, respectively. Furthermore, increasing salinity decreased such parameters significantly with respect to the studied amendments. Anthesis date, maturity date and plant height values decreased significantly, by 9.5 and 14.2%, 7.9 and 12.3% and 12.0 and 20.7%, respectively in the saline and highly saline soils, as compared with the non-saline soil (Table 1). Application of P fertilizers or organic amendments, or their combinations, alleviated such effects induced by salinity and deficit irrigation, enhancing phenology and plant height significantly. Treatment of Ps + FYM and Pa + FYM resulted in the highest values of anthesis, maturity and plant height by 6, 3.8 and 12.8%, respectively (Table 1). Consequently, the interactions between salinity and irrigation, salinity and amendments, irrigation and amendments as well as salinity, irrigation and amendments were significant, indicating the importance of improved management practices on wheat growth. Other growth variables (i.e. spikes number/m^2^, number of grains per spike and grain weight) were also significantly enhanced due to P fertilization and organic amendment, despite adverse effects of salinity and deficit irrigation (Supplementary Table S3). Grain and straw yield of wheat were affected significantly by salinity and deficit irrigation, which could be partially overcome by the proposed amendments (Table 2). Wheat grain yield decreased by 28.0 and 43.7%, respectively in saline and highly saline soils, as compared to non-saline soil. Moreover, deficit irrigation significantly reduced grain yield (by 20.5%), as compared to full irrigation. Application of P fertilizers and organic amendments not only encountered such impacts but improved all the yield traits over control (Table 2). Combinations of Ps + FYM or Pa + FYM showed the highest grain yield, increased significantly, by 89 and 74%, respectively as compared to untreated soils.

**Table 1:** Days to 50% anthesis, maturity and plant height of wheat as affected by irrigation regimes, phosphorus fertilizers and organic amendments in three soils with different salinity levels.

Irrigation (I)	Characters	Anthesis (DAS)				Maturity (DAS)				Plant height (cm)			
	Salinity (S_a_)	N-S	S	HS	Mean	N-S	S	HS	Mean	N-S	S	HS	Mean
	Amendments (A)												
FI	Control	105.17	92.50	87.67	95.11	149.34	138.83	131.84	140.00	128.42	111.77	96.73	112.31
	P_s_	106.50	95.50	89.67	97.22	151.00	140.34	134.33	141.89	134.80	120.12	107.57	120.83
	P_a_	105.34	96.00	91.67	97.67	156.67	140.84	133.33	143.61	135.50	122.97	108.47	122.31
	SS	105.34	95.84	91.33	97.50	151.17	140.50	134.00	141.89	137.19	119.62	108.30	121.70
	P_s_ + SS	108.67	97.33	93.00	99.67	151.67	143.33	135.84	143.61	137.35	118.40	110.60	122.12
	P_a_ + SS	108.00	97.84	93.33	99.72	153.33	143.33	136.34	144.33	140.57	117.51	110.63	122.90
	FYM	105.50	96.17	91.00	97.56	151.34	140.83	133.50	141.89	135.10	122.75	110.63	122.83
	P_s_ + FYM	110.00	101.34	94.33	101.89	155.00	145.67	138.50	146.39	144.06	128.00	114.90	128.99
	P_a_ + FYM	108.17	98.34	94.33	100.28	153.17	144.17	136.50	144.61	138.85	124.17	110.63	124.55
DI	Control	101.17	90.33	86.00	92.50	144.17	131.33	125.67	133.72	113.57	100.49	86.61	100.22
	P_s_	101.83	93.34	87.83	94.33	146.33	134.34	128.00	136.22	119.65	103.87	89.27	104.26
	P_a_	102.00	92.50	87.50	94.00	147.00	133.17	126.75	135.64	121.78	108.08	98.73	109.53
	SS	100.84	92.67	88.17	93.89	145.67	133.34	127.17	135.39	119.48	107.96	95.82	107.75
	P_s_ + SS	103.50	94.00	87.83	95.11	148.00	136.00	129.67	137.89	120.36	108.04	98.71	109.04
	P_a_ + SS	103.17	94.17	88.33	95.22	148.83	135.34	128.50	137.56	121.11	105.21	93.27	106.53
	FYM	100.67	92.50	88.17	93.78	145.50	133.00	127.17	135.22	120.86	104.14	96.77	107.26
	P_s_ + FYM	105.00	93.17	89.50	95.89	149.67	136.83	130.67	139.06	125.19	108.93	100.52	111.55
	P_a_ + FYM	104.34	92.84	87.83	95.00	147.84	134.50	127.67	136.67	122.97	108.11	98.26	109.78
	Mean	104.73	94.80	89.86		149.76	138.09	131.41		128.71	113.34	102.02	
LSD ≤ 0.05	S_a_	2.11				4.06				5.74			
	I	2.71				4.13				6.04			
	A	3.27				5.78				7.12			
	S_a_ × I	5.72				8.71				12.74			
	S_a_ × A	5.16				9.11				11.23			
	I × A	4.62				8.17				10.07			
	S_a_ × I × A	7.29				12.89				15.87			

Note, FI and DI: full and deficit irrigation respectively, N-S: non-saline soil, S: saline soil, HS: high saline soil, Ps: superphosphate fertilizer, Pa: ammonium phosphate, SS: sewage sludge, FYM: farmyard manure, S_a_: salinity factor, I: irrigation, A: amendments. Data represent the average of the two growing seasons.

**Table 2:** Wheat grain and straw yields as affected by irrigation regimes, phosphorus fertilizers and organic amendments applied to three soils with different salinity levels.

Irrigation (I)	Characters	Grain yield (t ha^−1^)				Straw yield (t ha^-1^)			
	Salinity (S_a_)	N-S	S	HS	Mean	N-S	S	HS	Mean
	Amendments (A)								
FI	Control	5.150	3.894	2.950	3.998	6.637	5.217	3.617	5.157
	P_s_	6.029	4.118	3.530	4.559	7.893	5.844	4.764	6.167
	P_a_	5.785	4.482	3.485	4.584	8.078	6.820	5.173	6.690
	SS	5.553	4.357	3.385	4.432	9.638	8.550	5.983	8.057
	P_s_ + SS	8.999	6.756	4.951	6.902	10.259	9.206	7.550	9.005
	P_a_ + SS	9.297	7.058	5.151	7.169	10.834	9.802	7.668	9.435
	FYM	6.104	4.519	3.232	4.618	9.292	7.504	5.862	7.553
	P_s_ + FYM	9.456	7.405	5.497	7.453	10.959	9.563	8.253	9.592
	P_a_ + FYM	8.901	6.668	4.918	6.829	10.745	8.621	7.065	8.810
DI	Control	4.158	2.946	2.343	3.149	5.750	4.153	2.604	4.169
	P_s_	5.177	3.718	3.255	4.050	6.585	5.511	4.025	5.374
	P_a_	4.800	3.309	2.836	3.648	6.352	5.118	4.193	5.221
	SS	4.484	3.111	2.919	3.505	6.673	5.276	3.490	5.146
	P_s_ + SS	6.261	4.539	3.402	4.734	7.138	5.425	4.068	5.544
	P_a_ + SS	7.042	4.438	3.481	4.987	8.372	5.647	4.290	6.103
	FYM	4.846	3.269	2.936	3.684	7.456	4.365	3.483	5.101
	P_s_ + FYM	7.288	4.364	3.844	5.165	8.433	5.655	4.385	6.158
	P_a_ + FYM	6.909	4.377	3.356	4.881	7.830	5.315	3.552	5.566
Mean		6.458	4.629	3.637		8.274	6.533	5.001	
LSD ≤ 0.05	S_a_	0.206				0.248			
	I	0.114				0.167			
	A	0.236				0.305			
	S_a_ × I	0.241				0.351			
	S_a_ × A	0.372				0.480			
	I × A	0.334				0.431			
	S_a_ × I × A	0.526				0.679			

Note, FI and DI: full and deficit irrigation respectively, N-S: non-saline soil, S: saline soil, HS: high saline soil, Ps: superphosphate fertilizer, Pa: ammonium phosphate, SS: sewage sludge, FYM: farmyard manure, S_a_: salinity factor, I: irrigation, A: amendments. Data represent the average of the two growing seasons.

In addition, the interactions between SS and FYM either added solely or combined with P fertilizer were non-significant. On the other hand, the interaction between all the treatments (i.e. salinity, irrigation and amendments) was significant for wheat grain and straw yield. Beside wheat growth and yield components, application of P fertilizers (i.e. Ps and Pa) and organic amendments (i.e. SS and FYM) and combinations of them, significantly improved quality traits (i.e. protein and carbohydrates), indicating the importance of these treatments in food security and nutrition value (Table 3). Application of Ps + FYM and Pa + FYM resulted in the highest content of protein and carbohydrates followed by Ps + SS and Pa + SS without significant differences between them. The quality traits in non-saline soil and received full irrigation were higher than those in saline and highly saline soils or received deficit irrigation. Nevertheless, application of P fertilizers and organic amendments could close the gap induced by salinity and deficit irrigation and increase protein and carbohydrate contents in wheat grains (Table 3).

**Table 3:** Quality traits of wheat grains as affected by irrigation regimes, phosphorus fertilizers and organic amendments applied to three soils with different salinity levels.

Irrigation (I)	Characters	Protein (%)				Carbohydrates (%)			
	Salinity (Sa)	N-S	S	HS	Mean	N-S	S	HS	Mean
	Amendments (A)								
FI	Control	9.52	8.41	7.87	8.60	74.71	70.43	68.31	71.15
	Ps	9.83	9.00	8.49	9.11	76.38	71.66	69.30	72.45
	Pa	9.93	9.24	8.92	9.36	76.00	72.42	68.64	72.35
	SS	10.23	9.10	8.34	9.22	75.33	71.88	69.65	72.29
	Ps + SS	11.40	10.55	9.75	10.57	77.50	73.89	69.29	73.56
	Pa + SS	12.22	10.92	9.87	11.00	78.87	73.54	71.50	74.64
	FYM	10.17	7.52	8.73	8.81	76.25	72.68	71.24	73.39
	Ps + FYM	12.91	11.47	10.15	11.51	80.07	74.50	72.72	75.76
	Pa + FYM	12.12	11.34	9.55	11.00	76.97	70.67	69.76	72.47
DI	Control	9.07	8.26	7.29	8.21	72.22	68.74	67.12	69.36
	Ps	9.63	8.82	8.13	8.86	72.93	69.77	67.37	70.02
	Pa	9.92	8.88	8.40	9.07	73.60	71.04	71.13	71.92
	SS	10.11	9.06	8.61	9.26	73.04	70.38	70.18	71.20
	Ps + SS	11.12	9.48	8.73	9.78	72.84	70.72	69.17	70.91
	Pa + SS	11.03	9.33	8.95	9.77	73.01	70.84	70.23	71.36
	FYM	10.66	8.72	8.40	9.26	74.14	72.12	70.34	72.20
	Ps + FYM	12.19	10.15	8.86	10.40	75.43	73.33	71.41	73.39
	Pa + FYM	11.99	9.73	8.70	10.14	73.49	70.36	69.57	71.14
Mean		10.78	9.44	8.76		75.15	71.61	69.83	
LSD ≤ 0.05	S_a_	0.53				2.08			
	I	0.37				1.73			
	A	0.67				2.13			
	S_a_ × I	0.79				3.65			
	S_a_ × A	1.05				3.36			
	I × A	0.94				3.01			
	S_a_ × I × A	1.48				4.75			

Note, FI and DI: full and deficit irrigation respectively, N-S: non-saline soil, S: saline soil, HS: high saline soil, Ps: superphosphate fertilizer, Pa: ammonium phosphate, SS: sewage sludge, FYM: farmyard manure, S_a_: salinity factor, I: irrigation, A: amendments. Data represent the average of the two growing seasons.

## Discussion

The corresponding reduction in soil pH with SS and FYM treatments may be ascribed to the production of humic acids and organic carbon biodegrades. Application of organic amendments, particularly SS, increased soil salinity and sodicity, which may be attributed to the high content of salts and alkaline substances in SS. Nevertheless, application of SS alone or combined with P fertilizers increased OM and AW in soil, followed by FYM, due to the input of OM and nutrients from the organic amendments (Supplementary Table S2). Increased OM in soil, in turn, improved other soil properties^20^, such as increased water holding capacity and available water, even in the highly saline soil. Consequently, the hazard effect of DI was alleviated. Conversely, application of SS resulted in the highest values of soil bulk density and lower values of hydraulic conductivity, due to higher SAR content in SS (Supplementary Table S2). Increased SAR from SS application may have caused dispersion of soil aggregates and subsequent decrease of porosity. In contrast, application of FYM improved soil physical properties (i.e. bulk density and hydraulic conductivity), as indicated by the lowest value of Bd and higher value of HC. This can be attributed to high OM and low Na^+^ content in FYM (Supplementary Table S2)^21^. Regardless of soil amendments, increasing soil salinity as shown in highly saline soil had an adverse effect on soil bulk density, HC, OM and AW. In addition to SAR^21^, salinity may also have an adverse effect on soil microbial activity^22^. Interestingly, application of organic amendments, particular FYM, alleviated the effects of salinity and DI on soil physical and chemical properties due to input of organic matter, which improved leaching process in saline soils. Therefore, organic matter content is the key for enhancing Na^+^ leaching and improving soil quality.

Deficit irrigation and salinity are the critical factors that negatively affect soil fertility and crop production in the arid and semiarid regions. They significantly decreased soil available macro nutrients (i.e. N, P and K), due to higher salinity caused by less irrigation than evapotranspiration, particularly in the highly saline soil. Consequently, highly saline soils occur frequently in combination with DI and drought, thus causing negative impacts on microbial activity^23^, organic matter decomposition^24^, and decreased availability of N, P and K in soil. Application of P fertilizers combined with organic amendments is considered a successful management tool, improving the availability of soil macronutrients, especially under salinity or drought stress. Furthermore, organic amendments, particularly SS, reduced soil pH, and thus increased the availability of macronutrients. However, application of organic amendments combined with P fertilizers also increased total content of heavy metals (i.e. Cd, Ni and Pb), particularly SS, due to high content of such metals in SS. Nevertheless, total contents of Cd and Ni in the soils received SS combined with P fertilizers were lower than the threshold values according to limits described by Tóth *et al*.^25^. In comparison, total content of Pb in all the studied soils was above the threshold limit regardless of amendments application or not. This contamination problem requires a proper remediation program. These findings highlight the importance of P fertilization and organic amendments for improving soil quality and crop production in this region.

**Figure 4:**
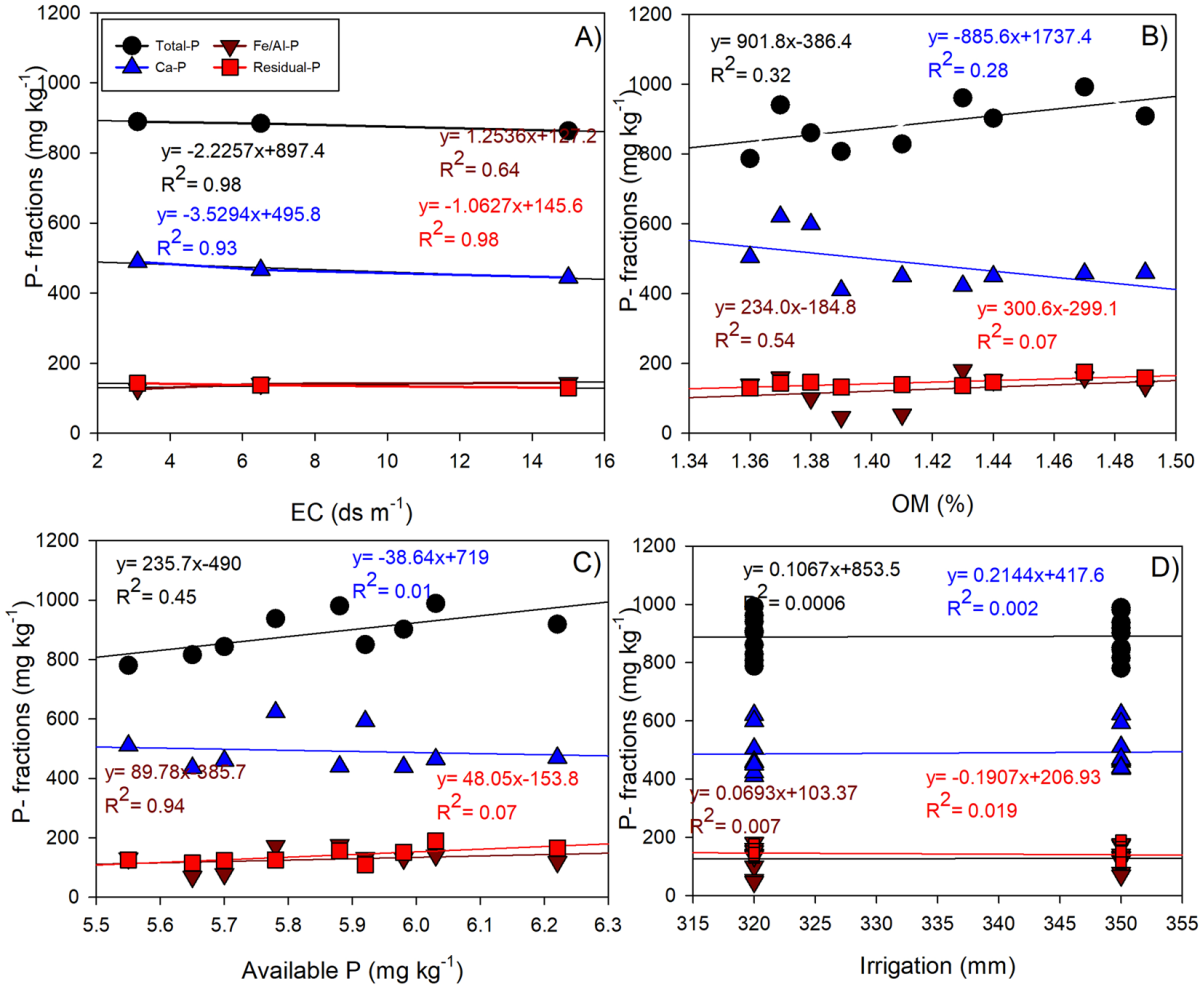
Relationship between soil salinity (**A**), organic matter (**B**), available P (**C**) and irrigation water applied (**D**), and soil P fractions. Irrigation water here represent only calculated irrigation water without precipitation.

The combined application of P fertilizers and organic amendments significantly increased P accumulation, which is in agreement with previous studies applied with cattle manure and P fertilizers^26,27^. This may be due to low levels of P in the soils and inputs from both organic and inorganic P sources^28^. Available P fraction in the studied soils represents only 0.89, 0.76 and 0.74% of the total P in non-saline, saline and highly saline soils, respectively (Supplementary Table S1). Combined application of P fertilizers and organic amendments showed a synergetic effect on soil available P, as compared to individual sources (Fig. 2B), which may be partially attributed to release of both P and low molecular weight organic acids from decomposition of organic amendments^14^. The organic acids/anions can dissolve insoluble P and compete with phosphate for adsorption sites on surfaces of soil particles, thus enhancing P availability^29^. Another mechanism could be due to enhanced microbial activity through dissolved organic carbon in soil^30^. The Ca-P was the most abundant fraction in the soils due to high content of Ca^2+^ that forms calcium phosphates with P. Unlike available P fraction, Ca-P decreased by application of organic amendments, likely due to calcium solubilization in response to decreased soil pH caused by organic acids released from organic matter decomposition. In comparison, application of P fertilizers without organic amendment resulted in an increase in the Ca-P fraction, indicating the importance of organic amendments in transforming Ca-P fraction to available-P pools. Compared with Ca-P, Fe/Al-P fraction was small, due to the nature of alkaline soils with limited amounts of active Fe and Al. Interestingly, this fraction is generally unstable due to the fluctuation of redox conditions according to soil conditions^31^. Application of P fertilizers increased Fe/Al-P fraction, likely due to adsorption of phosphate on Fe and Al oxides/hydroxides^32^. In this study, soil properties, irrigation regime and amendments significantly influenced soil P transformations (Figs. 3 and 4). Firstly, total P in soil tended to decrease with increasing salinity as indicated in the regression equations (Fig. 4A). Soil EC showed a significant negative relationship with total P (R^2^ = 0.98), Ca-P (R^2^ = 0.93), Fe/Al-P (R^2^ = 0.64) and residual-P fraction (R^2^ = 0.98). This may be due to the negative effect of salinity on soil physical, chemical and biological properties. By contrast, increasing soil organic matter showed a significant positive relationship with total P (R^2^ = 0.32), Fe/Al-P (R^2^ = 0.54) and residual-P (R^2^ = 0.07), whilst Ca-P fraction showed a negative relationship with soil organic matter (R^2^ = 0.28), (Fig. 4B). Soil available P showed a significant positive relationship with all fractions of P (Fig. 4C). By contrast, the effect of irrigation water applied on soil P fractions was non-significant (Fig. 4D), despite its effect on other soil properties (Figs. 1 and 2). In conclusion, the combined application of mineral P fertilizers with organic amendments, increased soil available P and other fractions due to several reasons: (1) the organic amendment adds P to the soil, (2) application of organic amendment combined with fertilizer P reduces the probability of P fixation in soils through adsorption and/or precipitation^33^, and (3) organic amendments mobilize native soil P^34^.

Soil salinity decreased yield and quality of wheat, due to its negative impact on soil available water and nutrients^6^. Similarly, water deficit during the sensitive growth stages (flowering and grain filling) caused a considerable reduction in wheat yield, and quality. This is mainly due to the negative effect of DI on plant physiological features such as accelerated leaf senescence, assimilate translocation, sink capacity, reduced grain set, as well as the oxidative damage to assimilatory machinery^35,36^. Consequently, the combined application of P fertilizers and organic amendments could alleviate the negative impacts of salinity and DI, improved soil physical and chemical properties, and thus enhanced wheat yield and quality. These treatments increased soil organic matter, available macronutrients and available water, and alleviated the hazardous impacts of salinity and deficit irrigation. Furthermore, soil P in the arid and semi-arid regions has very low availability due to high fixation, and slow diffusion, thus limiting plant growth and crop yield. Application of P fertilizers combined with organic amendments can significantly enhance soil P availability and increase wheat yield and quality.

## Materials and Methods

### Study sites and soil characteristics

Three field experiments were conducted on clay soils in Egyptian Nile delta with different ranges of electrical conductivity (EC) for two successive winter growing seasons 2016/2017 and 2017/2018. A new and high yielding wheat cultivar (*Triticum aestivum* L., cv Sakha 95) was selected for the experiments carried out in the three different locations, i.e. Kafrelsheikh (31.0N and 30.9 E), El-Riad (31.3N and 31.2 E), and Hamoul (31.4 N and 31.5 E), with non-saline (NS), saline (S) and highly saline (HS) soil, respectively (Supplementary Table S4). These locations were selected based on the variability of soil and irrigation water quality, representing different soil types in Egyptian Nile delta^37^. The average range of ground water table recorded (1.8–2.0 m), (1.6–1.8 m) and (1.2–1.4 m) for locations Kafrelsheikh (N-S), El-Riad (S) and Hamoul (HS) respectively^38^.The locations show a limited variation in climatic data through last three decades (1980–2010) as they were all located in the first agroclimatic zone in Egypt (Supplementary Fig. S1). However, there is a little variation of climatic data among these locations during wheat growing seasons (Table 4). Soil samples were taken at different depths (0–45 cm) from all the locations for physical and chemical analysis (Supplementary Table S4). The preceding crop in all the locations was maize in the first growing season (2016/2017) and rice in the second growing season (2017/2018). Wheat seeds were sown on 15^th^ November for all the locations at the rate of 140 kg ha^−1^ and harvested on 15^th^ April in both seasons.

**Table 4:** Weather situations in the studied locations during two growing seasons, season 1 (2016/2017) and season 2 (2017/2018) from sowing (November 15) to harvest (April 15).

Weather parameters and units	N-S (KFS location)		S (El-Riad location)		HS (Hamoul location)	
	Season 1	Season 2	Season 1	Season 2	Season 1	Season 2
TMAX (°C)	20.0	22.2	20.5	22.5	20.2	22.0
RH2M (%)	65.9	64.1	64.5	63.8	64.9	64.8
TMIN (°C)	12.0	13.4	11.9	13.2	12.5	13.9
RAIN (mm)	150.4	72.5	123.6	71.9	115.6	73.4
TDEW (°C)	8.9	10.2	8.7	10.1	9	10.6
WIND (m/s)	3.4	3.3	3.4	3.2	3.6	3.4
SRAD (MJ/m2/day)	13.4	13.2	13.6	13.3	13.6	13.3

Locations N-S, S and HS represent non-saline, saline and highly saline soils respectively. Parameters TMAX, RH2M, TMIN, WIND and SRAD represent average values of maximum temperature, relative humidity, minimum temperature, dew/frost point, wind speed and solar radiation through growing season. Meanwhile, values of RAIN represent total accumulated precipitation (mm).

### Agricultural practices and experimental design

The experiment in each location was laid out in a split plot design with three replicates. The tillage system included ploughing the soil up to 30 cm depth using a country plough. The plot size was 40 m^2^ (8 m × 5 m), with 0.5 m alley surrounding each plot. The main treatments included irrigation scheduling at different levels of depletion from soil available water to be 50% (full irrigation, FI) and 70% (deficit irrigation, DI). Soil moisture content was monitored daily using time-domain reflectometry (TDR), to specify the irrigation timing in both treatments. The main bases of FI and DI treatments had been defined according to the change in depletion of soil available water for each soil type. The FI represents the optimum irrigation design in clay soil and could be achieved by triggered irrigation at 50% depletion from soil available water (readily available water). At this point, crop could not expose to deficit irrigation, as irrigation triggered at readily available water. As the available water content differs between soil types, Supplementary Table S4, irrigation timing with FI treatment triggered when soil moisture reached 31.0, 30.1 and 29.0% for N-S, S and HS soils respectively. These values were defined by multiplying soil available water in each soil Supplementary Table S4 by required depletion (50%) and extracted the product from soil field capacity in each soil type. To convert these values of soil moisture to irrigation timing, the calibrated TDR had been used to daily monitor soil moisture until reaching previous values of moisture content. The day we reach this content of moisture is the day of triggered irrigation. To quantify the irrigation quota, we conducted the current soil moisture to field capacity to each soil again using the following equation:

1 $$AW=\frac{SM1-SM2}{100}\times BD\times D\times A$$

where: AW = Applied water in m^3^, SM1 = Soil moisture at field capacity (%), SM2 = Soil moisture before next irrigation based on the required depletion (%), BD = Soil bulk density (Mg m^−3^), D = Soil depth of the effective root zone (m), and A = Irrigated area (m^2^)

For controlling in water applied to each treatment and soil, a rectangular sharp crested weir had been used through the following equation:

2 $$Q=CL{(H)}^{1.5}$$

where: Q = Water discharge (m^3^ S^−1^), C = Empirical coefficient determined from discharge measurement, 0.3, L = Length of the crest (m), H = Head above the weir (m)

The same procedures of irrigation scheduling (timing and quantity) were used with deficit irrigation, considering the required depletion to be 70% from soil available water.

The sub main treatments consisted of control (without addition), superphosphate (Ps, 120 kg ha^−1^), ammonium phosphate (Pa, 120 kg ha^−1^), farmyard manure (FYM, 10 t ha^−1^), sewage sludge (SS, 5 t ha^−1^), Ps + FYM, Ps + SS, Pa + FYM and Pa + SS. Phosphorus fertilizers and organic amendments were applied to soil with tillage. The chemical characteristics and heavy metal content of FYM and SS are shown in Supplementary Table S3. The FYM is the decomposed products of animal excreta (i.e. dungs and urines) with some crop residues such as rice and cotton straw. Sewage sludge collected from a domestic effluent treatment plant (Messer wastewater treatment plant, Kafrelsheikh), which received sludge from both domestic and industrial sources with dominant of domestic and treated first before applying into farmland. Dewatering and storage process used for treatments, while conditioning the sludge with lime followed by dewatering and storage for 5 months before application. Sugar beet factory lime (SBFL), produced during sugar purification process from Delta Sugar Factory (Hamoul, Egypt) mixed with sludge at rate of 10%. Potassium fertilizer was added to all the experiments in one dose prior to the first irrigation at the rate of 60 kg K_2_O ha^−1^ as potassium sulphate (containing 48% K_2_O). Nitrogen fertilizer as urea was added to all the experiments in two equal doses (180 kg N ha^−1^), the first dose was applied with the first irrigation and the second dose was added with the second irrigation. The experiments in the three locations, irrigated with good quality water from canals branched from the River Nile with EC values (0.7, 0.9 and 1.2 dS m^−1^) for Kafrelsheikh, El-Riad and Hamoul locations, respectively. Detailed information of irrigation water scheduling (timing and quota) and subsequent total irrigation water amount for each irrigation regime are explained in Supplementary Table S5. For weed control, two chemical herbicides were used at different stages during crop growth period. These chemicals included Granstar 75% DF (Tribenuron-methyl) at rate of 19.2 g ha^−1^ and applied at 25 days after sowing (DAS), as well as Topic 15% WP (Clodinafop-propargyl) at rate of 350 g ha^−1^, applied at 45 DAS.

## Measurements

### Soil physical and chemical properties

Soil chemical properties before cultivation and after harvesting from all the locations were analyzed using the classical methods^39,40^. Undisturbed soil samples were obtained for measuring soil physical properties^41,42^. Soil moisture parameters such as field capacity (FC) and permanent wilting point (PWP) were measured using pressure membrane apparatus at the pressures of 0.3 and 15 bar, respectively. Saturated hydraulic conductivity was determined in field using Guelph permeameter apparatus^43^. Soil available N, P and K were extracted and determined with the standard methods^44–46^. Additional part of soil samples was digested for total heavy metal analysis (Cd, Ni and Pb) using inductively coupled plasma-optical emission spectrometry (Ultima2, Horiba Scientific, Germany)^47^. In subsamples soil P fractionation was determined following the modified sequential extraction method^48,49^. Specifically, air dried soil (1 g) was extracted with NaHCO_3_, giving the available fraction of P. The soil residue was then treated with NaOH and shaken for 16 h and the extract was analyzed for Fe/Al-P fraction. The soil residue was then extracted with HCl for 16 h to obtain Ca-P fraction. Finally, the residual P fraction was obtained by digesting the remaining soil with concentrated HNO_3_. Following each extraction, soil residues were separated from extraction solution, through centrifuging and filtering the supernatant with filter papers and determining P by the ascorbic acid reduction-molybdenum colorimetric method.

### Yield, yield attributes and quality of wheat

Phenology parameters such as anthesis and maturity dates, plant height, number of spikes per m^2^, number of grains per spike, 1000-grain weight, grain yield (dried in oven at 70 °C for 24 h), and straw yield were measured in all the experiments. Plant grains of ten wheat plants from each experimental plot, were oven dried at 70 °C for 48 h, then ground to fine powder for quality analysis. Protein in wheat grains was calculated by multiplying the N content determined in grains^50^ by 5.7, meanwhile, total carbohydrate was measured in the ground powder as described by^51^.

### Statistical analysis

The obtained data were statistically analyzed using the analysis of variance (ANOVA) according to^52^, using PASW statistics 21.0 (IBM Inc., Chicago, IL, USA). Treatment means were compared using the least significant difference test (LSD) at P < 0.05. Combined analysis for the two seasons and all three locations was performed using the homogeneity test.

## Conclusions

Application of P fertilizers and organic amendments to soils resulted in significant improvement in soil physical and chemical properties, enhanced wheat yield and quality traits. The combined treatments of P fertilizers with sewage sludge or farmyard manure decreased soil salinity, soil bulk density, whilst increased organic matter content, hydraulic conductivity, available water and macronutrients. Environmentally, application of sewage sludge and/or farmyard manure increased total content of heavy metals (Cd, Ni and Pb) in soil. Notwithstanding, the levels of total Cd and Ni in soil are generally below the critical limits for ecological and health risk. However, the levels of total Pb in soil exceeded the hazardous limits, which requires additional program for remediation. Although P is a very important element for plant growth, the soils in the arid and semi-arid regions are generally low in available P. Combination of different sources of mineral P with organic amendments can significantly increase soil available P regardless of soil types and irrigation regimes.

## Supplementary information

Supplemental Information.
