# Supplementary material for: The integrated effect of salinity, organic amendments, phosphorus fertilizers, and deficit irrigation on soil properties, phosphorus fractionation and wheat productivity
Source: Sci Rep. 2020 Feb 17;10:2736. doi: 10.1038/s41598-020-59650-8 (PMC7026061; doi:10.1038/s41598-020-59650-8)
Supplement: Supplementary file 1 — Supplemental Information. [file 41598_2020_59650_MOESM1_ESM.pdf]

The integrated effect of salinity, organic amendments, phosphorus fertilizers, and deficit irrigation on soil properties, phosphorus fractionation and wheat productivity

Zheli Ding[1], Ahmed M.S. Kheir[1,2*], Marwa G.M. Ali[2], Osama A.M. Ali[3], Aly I.N. Abdelaal[2], Xin'e Lin[1], Zhaoxi Zhou[1], Bizun Wang[1], Beibei Liu[4], Zhenli He[5]

[1]Haikou Experimental Station, Chinese Academy of Tropical Agricultural Sciences (CATAS), Haikou, China

[2]Soils, Water and Environment Research Institute, Agricultural Research Center, Giza, Egypt

[3]Crop Science Dept., Faculty of Agriculture, Menoufia University, Shebin El-Kom, Egypt

[4]Institute of Environmental and Plant Protection/Environmental Impact Assessment and Risk Analysis Center, Chinese Academy of Tropical Agricultural Sciences, Haikou, Hainan, China

[5]University of Florida, Institute of Food and Agricultural Sciences, Indian River Research and Education Center, Fort Pierce, FL 34945, USA

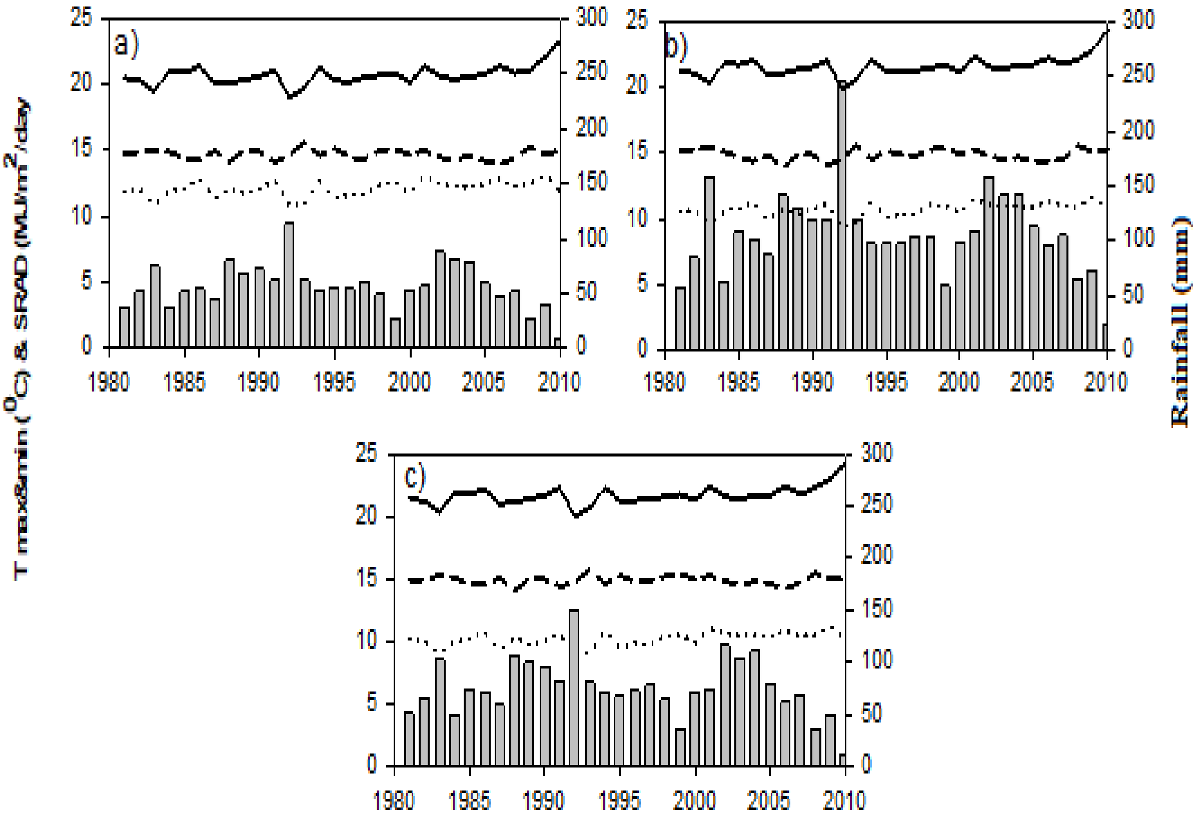

Supplementary Figure. S1 Weather data represented as lines (maximum temperature), dashed lines (solar radiation), dotted lines (minimum temperature) and bars (rainfall) of wheat during the growing season (planting-harvest) for 30 years (1981-2010) in the three studied locations. The locations are a, b, and c, for Kafrelsheikh, El-Riad, and Hamoul, respectively represent the first agroclimatic zone in Egypt.

Treatments	Non-saline soil	Saline soil	Highly saline soil
Control	7.78abc	8.00abc	8.18a
Ps	7.79abc	8.12abc	8.19a
Pa	7.77abc	8.10abc	8.16ab
SS	7.52c	7.78c	8.00abc
Ps+SS	7.58abc	7.95abc	8.14ab
Pa+SS	7.55bc	7.80bc	8.10abc
FYM	7.60bc	7.85bc	8.12abc
Ps+FYM	7.70abc	7.99abc	8.18a
Pa+FYM	7.66abc	7.99abc	8.17ab

Supplementary Table S1 Soil pH subjected to organic amendments and P fertilizers in different soil types. Note: FYM, farmyard manure, SS: sewage sludge, Ps: single superphosphate, Pa: di-ammonium phosphate. Values accompanied by different letters within the same column are significantly different at the $P < 0.05$ level of significance.

Irrigation (I)	Characters	Spikes number m^{-2}				Number of grains $spike^{-1}$				1000-grain weight (g)			
	Salinity (S_a) Amendments (A)	N-S	S	HS	Mean	N-S	S	HS	Mean	N-S	S	HS	Mean
FI	Control	338.00	317.67	290.50	315.39	53.83	45.50	40.00	46.44	42.85	40.77	39.25	40.96
	P_s	352.34	335.67	328.50	338.84	60.00	50.67	45.67	52.11	43.79	42.12	40.10	42.00
	P_a	349.50	334.17	327.34	337.00	58.67	52.33	45.17	52.06	43.51	41.97	39.99	41.82
	SS	346.33	330.67	328.34	335.11	59.84	51.84	44.83	52.17	43.68	41.62	39.74	41.68
	P_s+SS	388.00	368.34	348.50	368.28	61.00	54.50	47.67	54.39	47.85	45.39	42.25	45.16
	P_a+SS	385.17	356.00	348.33	363.17	60.17	52.00	48.00	53.39	47.07	44.15	41.93	44.38
	FYM	345.50	331.67	325.67	334.28	57.33	49.67	44.83	50.61	43.10	41.40	39.62	41.37
	P_s+FYM	393.50	370.50	355.17	373.06	67.84	61.83	53.84	61.17	47.97	45.05	42.48	45.17
	P_a+FYM	389.67	366.67	351.67	369.34	60.17	56.17	50.34	55.56	47.30	44.67	41.67	44.55
DI	Control	323.33	297.17	270.17	296.89	48.34	41.50	36.33	42.06	42.55	40.42	38.82	40.60
	P_s	333.67	312.17	297.34	314.39	50.50	44.34	40.67	45.17	43.17	40.97	39.29	41.14
	P_a	330.00	304.34	286.34	306.89	49.84	45.33	39.67	44.95	42.80	40.99	39.24	41.01
	SS	326.83	303.67	291.67	307.39	48.50	44.67	39.50	44.22	42.48	40.92	38.72	40.71
	P_s+SS	346.84	331.17	313.50	330.50	53.17	50.17	47.17	50.17	44.49	43.67	41.40	43.19
	P_a+SS	349.84	322.34	297.34	323.17	51.33	47.00	41.17	46.50	44.79	42.79	39.38	42.32
	FYM	325.34	304.84	291.84	307.34	51.50	44.67	40.83	45.67	42.34	41.04	39.25	40.88
	P_s+FYM	360.83	324.50	308.50	331.28	55.17	50.00	43.50	49.56	45.43	42.35	40.36	42.71
	P_a+FYM	351.34	314.50	303.50	323.11	50.17	45.17	42.67	46.00	45.14	41.45	39.39	41.99
	Mean	352.00	329.23	314.68		55.41	49.30	43.99		44.46	42.32	40.16	
LSD ≤ 0.05	S_a	11.51				3.57				1.92			
	I	10.64				4.08				1.15			
	A	15.57				4.95				NS			
	$S_a \times I$	22.45				8.61				2.43			
	$S_a \times A$	24.55				7.80				5.16			
	$I \times A$	22.02				7.00				4.62			
	$S_a \times I \times A$	34.71				11.04				7.29			

Supplementary Table S2. Wheat spikes number, grains number per spike and grain weight as affected by irrigation regimes, phosphorus fertilizers and organic amendments applied to three soils with different salinity levels. Note, FI and DI: full and deficit irrigation respectively, N-S: non-saline soil, S: saline soil, HS: high saline soil, Ps: superphosphate fertilizer, Pa: ammonium phosphate, SS: sewage sludge, FYM: farmyard manure, S_a: salinity factor, I: irrigation, A: amendments. Data represent the average of the two growing seasons.

Item	FYM	SS	Ps	Pa
EC (dS m^{-1})	2.3±0.05	2.8±0.04	n.d	n.d
SAR	5.00±0.1	20.0±0.2	n.d	n.d
pH	7.6±0.01	6.3±0.01	8.0±0.02	7.8±0.02
OM (%)	45±0.2	65±0.3	n.d	n.d
TN (mg kg^{-1})	3.7±0.1	4.1±0.2	1.2±0.01	1.8±0.01
TP (mg kg^{-1})	11±0.2	13±0.3	15.5	46.0
TK (mg kg^{-1})	40±0.5	50±0.6	n.d	n.d
Cd (mg kg^{-1})	4±1.0	75±5.0	4±0.01	1.8±0.02
Ni (mg kg^{-1})	14±3.0	138.7±5.5	1.2±1.0	0.8±0.5
Pb (mg kg^{-1})	60±4.1	671±15.0	3.5±2.5	1.8±0.7

Supplementary Table S3 Chemical characteristics and heavy metals content of soil organic amendments and phosphate fertilizers. Note: FYM, farmyard manure, SS: sewage sludge, Ps: single superphosphate, Pa: di-ammonium phosphate, EC: electrical conductivity, OM: organic matter content, TN: total nitrogen, TP: total phosphorus, TK: total potassium, n.d: non-detected, Means ± Stdev, n=3.

Item	Unit	Non-saline soil	Saline soil	Highly saline soil
EC (soil paste extract)	dS m^{-1}	3.1	6.5	15.0
pH (1:2.5 soil water suspension)		7.8	8.1	8.2
SAR		8.0	11.0	14.0
CEC	Cmol kg^{-1}	38.0	40.0	32.0
OM	%	1.45	1.50	1.32
Available N	mg kg^{-1}	55.0	50.0	38.0
Available P	mg kg^{-1}	6.5	6.0	5.2
Available K	mg kg^{-1}	220	200	185
Total P	mg kg^{-1}	787.3	785.0	695.2
Silt	%	29.7	25.5	20.5
Clay	%	51.9	52.3	57.6
Sand	%	18.4	22.2	21.9
Texture		Clay	Clay	Clay
Bulk density	Mg m^{-3}	1.20	1.28	1.35
Hydraulic conductivity	cm day^{-1}	2.5	2.2	2.0
Field capacity	%	41.0	40.0	38.5
Wilting point	%	21.0	20.2	19.5
Available water	%	20.0	19.8	19.0
Total Cd	mg kg^{-1}	2.5	3.1	3.0
Total Ni	mg kg^{-1}	35	42	40
Total Pb	mg kg^{-1}	280	300	270

Supplementary Table S4 Initial physical and chemical properties of non-saline, saline and highly saline soils in the three studied locations prior to treatments. Note: Data of each item is the average of three soil depths, EC: electrical conductivity, SAR: sodium adsorption ratio, CEC: cation exchange capacity

Soil types	N-S				S				HS			
Growing seasons	Season 1		Season 2		Season 1		Season 2		Season 1		Season 2	
Irrigation regime	Date	Quota	Date	Quota	Date	Quota	Date	Quota	Date	Quota	Date	Quota
Deficit irrigation	Nov, 15	90	Nov, 15	88	Nov, 15	95	Nov, 15	98	Nov, 15	102	Nov, 15	105
	Dec, 5	55	Dec, 8	65	Dec, 1	60	Dec, 3	65	Dec, 1	75	Dec, 2	70
	Jan, 15	80	Jan, 10	75	Jan, 5	70	Jan, 11	72	Dec, 31	83	Jan, 2	90
	March, 10	55	March, 8	92	March, 1	75	Feb, 27	85	Feb, 28	80	Feb, 25	95
Irrigation water applied (mm)		280		320		300		320		340		360
Rainfall (mm)		150.4		72.5		123.6		71.9		115.6		73.4
Total water applied (mm)		430.4		392.5		423.6		391.9		455.6		433.4
Full irrigation	Nov, 15	90	Nov, 15	88	Nov, 15	95	Nov, 15	98	Nov, 15	102	Nov, 15	105
	Dec, 1	40	Dec, 3	50	Nov, 28	53	Nov, 29	55	Nov, 25	65	Nov, 27	62
	Dec, 30	65	Jan, 1	70	Dec, 25	55	Dec, 20	62	Dec, 15	72	Dec, 18	82
	Jan, 25	45	Feb, 2	55	Jan, 18	62	Jan, 20	80	Jan, 18	75	Jan, 15	88
	March, 15	60	March, 13	77	March, 10	55	March, 5	85	Feb, 28	56	March,1	53
Irrigation water applied (mm)		300		340		320		380		370		390
Rainfall (mm)		150.4		72.5		123.6		71.9		115.6		73.4
Total water applied (mm)		450.4		412.5		443.6		451.9		485.6		463.4

Supplementary Table S5. Irrigation scheduling for wheat during two growing seasons (2016/2017 and 2017/2018) in three soil types non- saline (N-S), saline (S) and highly saline (HS). Number of irrigations in deficit and full regime were four and five respectively. Total water applied calculated by sum of irrigation water applied and total accumulated rainfall through season. Irrigation scheduling (date and quota) was determined for different irrigation regimes (DI and FI) based on the required depletion of soil available water.
